# Identification of Cotton Leaf Lesions Using Deep Learning Techniques

**DOI:** 10.3390/s21093169

**Published:** 2021-05-03

**Authors:** Rafael Faria Caldeira, Wesley Esdras Santiago, Barbara Teruel

**Affiliations:** 1 Faculty of Agricultural Engineering of the University of Campinas, FEAGRI/UNICAMP, Campinas 13083-875, Brazil; barbarat@unicamp.br; 2 Institute of Agricultural Sciences of the Federal University of the Jequitinhonha and Mucuri Valleys, ICA/UFVJM, Unaí 38610-000, Brazil; wesley.santiago@ufvjm.edu.br

**Keywords:** artificial intelligence, convolutional neural networks, image processing, precision agriculture

## Abstract

The use of deep learning models to identify lesions on cotton leaves on the basis of images of the crop in the field is proposed in this article. Cultivated in most of the world, cotton is one of the economically most important agricultural crops. Its cultivation in tropical regions has made it the target of a wide spectrum of agricultural pests and diseases, and efficient solutions are required. Moreover, the symptoms of the main pests and diseases cannot be differentiated in the initial stages, and the correct identification of a lesion can be difficult for the producer. To help resolve the problem, the present research provides a solution based on deep learning in the screening of cotton leaves which makes it possible to monitor the health of the cotton crop and make better decisions for its management. With the learning models GoogleNet and Resnet50 using convolutional neural networks, a precision of 86.6% and 89.2%, respectively, was obtained. Compared with traditional approaches for the processing of images such as support vector machines (SVM), Closest k-neighbors (KNN), artificial neural networks (ANN) and neuro-fuzzy (NFC), the convolutional neural networks proved to be up to 25% more precise, suggesting that this method can contribute to a more rapid and reliable inspection of the plants growing in the field.

## 1. Introduction

Cotton is one of the most economically important crops used for the production of natural fibers in the world [1]. It is raised in some 100 countries around the world, and its cultivation occupies some 2.5% of the arable land of the world [2].

Data from the official Brazilian government organ reveal that in 2019/2020, a yield of 28.2 million tons of cotton were produced from some 17 million hectares [3]. Despite the continental size of Brazil, its cultivation is concentrated in the states of Mato Grosso and Bahia, hot and humid regions where intensive cultivation and large technological investment must overcome climatic conditions which favor epidemics of fungal diseases, especially the ramularia leaf spot [4].

Found in all producing regions of the world, the ramularia is one of the main diseases facing cotton plantations [5,6]. Along with the pests and diseases of ramulose, alternaria spot and mites, it is responsible for a huge impact on the Brazilian cotton crop, causing losses in productivity and the quality of the fiber and seeds [7].

One of the specificities of cotton culture in humid tropical conditions, are the creation of natural conditions causing the culture to suffer considerable range of pests and diseases, requiring ever more efficient solutions [8,9,10].

The adoption of conventional strategies for the control of pests and disease (treatment of the seeds, selection of resistant genotypes, control of the density and period of the year for planting, crop rotation and the use of biological agents), it was not enough. The only resource capable of effectively controlling the attack of pests and diseases is the preventive application of agrochemical products such as insecticides, acaricides and fungicides [4,11]. 

However, research has shown that the inadequate and indiscriminate use of such products has aggravated the occurrence of pests and diseases in agricultural areas [12,13]. For cotton growers, the discussion goes beyond biological aspects and advances to economic ones, since the need for repeated applications of such products and increases in dosage have elevated the cost of production to the point that the expenditure for pesticides has exceeded 50% of the cost of production for the past three seasons [14].

There is a certain consensus in the scientific literature that for more sustainable and competitive production, the use of such products should be limited, especially those utilized for the control of pests and agricultural diseases [15,16].

According to Ghaffar et al. [17], it is possible to increase the profits from raising cotton and guarantee sustainability if precision technologies of agriculture are adopted. The overlapping of productivity maps and soil fertility, monitoring via satellites and drones, or even the localized application of products can provide significant economic and environmental gains and even reduce the use of pesticides by more than 50% [18].

One of the fundamentals of localized management of agricultural crops is the identification of the variability of attributes [19]. For the cotton grower, few reports of geospatial evaluation of the distribution of diseases are available, principally due to the need for various preventive applications of pesticides to combat diseases, which are difficult to control, and the lack of tools to optimize the process of mapping in the field [5,11]. 

In the past decade, research utilizing remote sensing and drones has become quite popular for the mapping of agricultural pests and diseases [20,21], although even with the considerable scientific advances available, these tools still present cultural and commercial restrictions as to use. For programs of Integrated Management of Pests and Diseases (MIPD), the use of agrochemical products is still the main tool available for management of a farm, whether applied indiscriminately at variable rates.

Since the basis for MIPD is the identification of pests and diseases associated with the level of economic damage, the main requirements of the program include the availability of qualified personnel in quantity compatible with the size of the fields [22]. According to Barbedo [23], the bottleneck for strategies such as the MIPD is the process of identification of pests and diseases, since it depends directly on the level of expertise of the technician who is monitoring the fields.

In an attempt to reduce human subjectivity. computer systems for the identification of agricultural pests and diseases are under development and have received considerable attention in recent years. Since the alterations in biophysical and biochemical properties of the plants altered by pathogens, such as pigmentation/color and texture, are visible, it is possible to use techniques of image processing to diagnose pests and diseases [24,25]. Such automatized systems for the detection of plant diseases are designed to identify and classify diseases by harnessing the know-how of specialists in phytopathology in combination with the use of convolutional neural networks (CNN) to extract symptomatic lesions. This is important because interpretation of images is crucial.

In 2014, Revathi and Hemalatha [26] proposed a system using the data of color, texture and shape in the processing of images to identify leaf diseases. Image processing was also used by Abade et al. [27], although they complained that certain crops such as cereals and grains which are prevalent in the food chain and high in economic importance are often overlooked by traditional approaches for the detection of diseases. Brandão et al. [28] used measures of spectral reflection to evaluate the growth and productivity of the cotton crop. Using multispectral images obtained by an airplane, Yang et al. [29], implemented an algorithm to detect areas of cotton culture suffering from the presence of the fungus *Phymatotrichopsis omnivore*.

When using standard image processing algorithms, the greatest impact on the results is found to be a function of the quality, type and resolution of the image, as well as the type of descriptor of characteristics and classifier selected. This is why so many studies have been limited to images taken under controlled lighting conditions. The use of convolutional neural networks produces better results than those obtained from the traditional pipeline of image processing. Moreover, the recognition of leaf lesions based on techniques of image processing can contribute to a more rapid and reliable inspection of a cotton crop, thus helping in the obtention of maps distinguishing the location of healthy plants from those where agrochemical products should be applied at variable rates.

Convolutional neural networks represent an evolution of traditional artificial neural networks, since they have a greater capacicty for learning due to the representation of the data in the form of a hierarchy, with various levels of abstraction. When well trained, such methods prove to be quite precise for the classification of patterns. It is the struccture of the CNN which exerts the greatest influence on the performance in the extraction and classification of the resources of the image. The results of Abade et al. [27] show a significant improvement when CNNs are used for the identification of plant diseases; moreover, the accuracy is reasonable, even with a set of data composed of images captured in actual field environments. Barbedo [30] was able to identify plant diseases on the basis of leaf lesions and spots using deep learning techniques for various crops with an accuracy greater than 70%. Deebe and Amuthe [31] obtained an accuracy varying from 62 to 96% using different convolutional neural network architectures but reached the conclusion that the networks work better for a set of existing controlled data, since the precision was reduced for data collected under actual field conditions. Boulente et al. [32] affirm that CNNs furnish a noteworthy performance in tasks related to the classification and detection of diseases in crops, managing complex problems with difficult image condtions.

This paper explores the use of deep learning models for the identification of the lesions encountered on the leaves of cotton plants in images obtained under field conditions of uncontrolled lighting. 

## 2. Materials and Methods

This paper is based on a set of data obtained from the images obtained periodically of the crop of 2018 and 2019 from a commercial cotton plantation in the municipality of Unai, in the state of Minas Gerais. The data consist of 60,659 images containing information showing healthy and lesioned leaves, as well as background material, such as soil and straw. The images are stored in the RGB color space, with a resolution of 102 × 102 pixels, and with no control of lighting, angle, or focal distance. The randomness of these characteristics makes it crucial that the algorithms analyzed are robust and provide invariable results.

**Figure 1:**
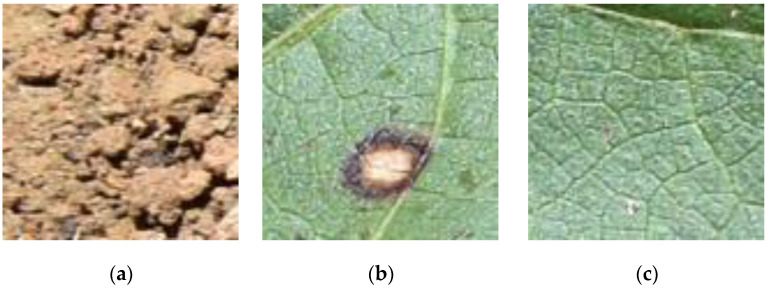
Examples of images of (**a**) background (soil), (**b**) lesioned leaf, and (**c**) healthy leaf.

To train and validate the algorithm, all images were analyzed in relation to visual quality and separated by specialized technicians into three classes: background (when 70% or more of the image was occupied by information about soil, residue, or other unknown element), healthy leaf (when 70% or more of the image was occupied by healthy leaf), and lesioned leaf (when 70% or more of the image was occupied by a leaf showing characteristics of attack by pests or disease) (Figure 1). This classification resulted in 39,215 background images, 6918 images of healthy leaves, and 11,722 of leaves with lesions. The images were then randomly separated into training and testing sets, composed of 70% and 30% of the images, respectively. The images were acquired using a digital Nikon camera (model D5500) set to automatic.

**Figure 2:**
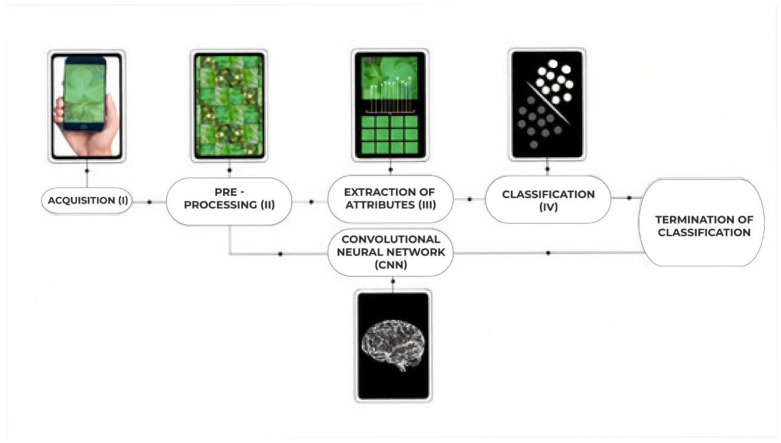
Pipeline adopted. In this paper, Steps III and IV were replaced by deep learning models.

The main algorithms for image processing for the detection of objects, plants, disease or pattern recognition can be resumed in the pipeline proposed by Gonzalez and Woods [33], starting with the acquisition of images and progressing with pre-processing, attribute extraction and classification, as shown in Figure 2.

In this paper, the processing pipeline included the following stages:

(I) Acquisition: Capture of cotton leaf images under natural field conditions, considering different phenological stages and different harvests.
(II) Pre-processing: Selection of images, removal of outliers and grouping by specialists into the respective classes
(III) Extraction of attributes: Extraction of the set of features of interest based on the statistical attributes of texture utilized by Pydipati et al. [34] and Bhimte et al. [35]. The set includes measures such as average level of grey, standard deviation, correlation, third moment, uniformity, and entropy. The equations for the statistical attributes of texture are presented in Table 1.
(IV) Classification: Four different machine-learning algorithms were tested: Artificial Neural Networks [36], Hybrid Neuro-Fuzzy networks [37], Support vector machines [38] and Closest k-Neighbors [39].

Since the objective of the paper is to compare the performance of the deep learning technique with different standard image processing algorithms, Convolutional Neural Networks (CNN) replaced Stages III and IV of the pipeline above (Figure 2). Given the characteristics of this model, it is assumed to be able to learn from the training set and satisfactorily identify lesions.

### Convolutional Neural Networks

Introduced in the area of machine learning in 1990 by Lecun et al. [40], CNNs represent the continuing development of traditional artificial neural networks; they have a greater capacity for learning due to the representation of the data in hierarchic form in various levels of abstraction.

**Figure 3:**
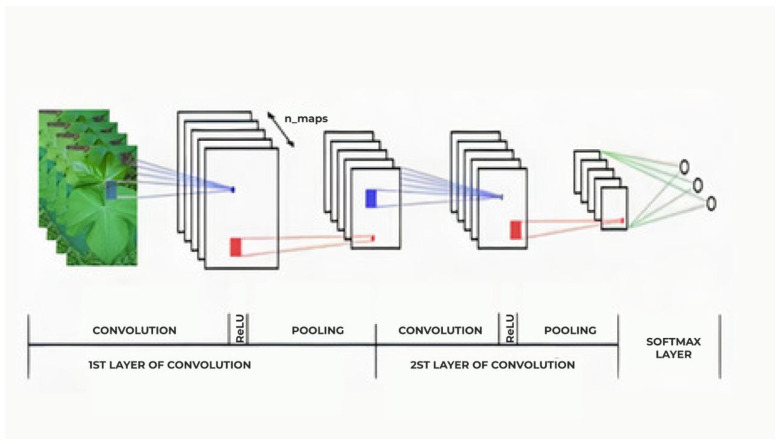
Diagram of a Convolutional Neural Network. (Reprinted with permission from ref. [42]).

Basically, a CNN functions by performing various convolutions in different layers of the network. This creates different representations of the set of learning data, starting with the most general in the first layers and becoming more specific in the deeper layers [41]. The convolutional layers act as a kind of extractor of characteristics, since the reduction of the dimensionality of the entrance data groups them in layers (Figure 3).

The convolutional layers codify various basic resources into more discriminative ones, creating a series of intermediate layers separated by rectified linear units (ReLU) and layers of enveloping (pooling). In a more generic explanation, the convolutional layers act as filters, which transform one entrance image into another, highlighting specific patterns [42]. In the next to last layer of the network, the final characteristics of the image are emphasized; thus, the final stage (layer) of the network acts as a linear classifier. A more detailed description of this kind of model can be found in Lecun et al. [43].

With standard image processing algorithms, the impact on the result occurs as a function of image quality, type and resolution; it also depends on the type of descriptor of the characteristics and the classifier selected. In the case of a CNN, it is the structure, which has the greatest influence on performance in the extraction and classification of the resources/aspects of the images [44]. Ososkov and Goncharov [45] mention that shallow networks limit the learning capacity because of the limited number of layers.

For the recognition of lesions on cotton leaves based on image processing, the basic architecture for a CNN is found with Google Net [46] and ResNet50 [47].

The processing structure of images for deep learning considers a stage for the weighting of the data and another for the artificial increase of data. Once the data is separated into training and testing sets, the number of images in the class with the smallest number of images is defined as the maximum value for the counting of images in each class for each of the two sets of data (training or test) [48].

The final step involves the artificial increase of the data, with images redimensioned to a standard size and defined for the first layer of the CNNs, i.e., the images were redimensioned to 224 × 24 × 3 pixels.

Both the standard image processing algorithms and CNNs, as well as the training and testing processes, were implemented using the Toolbox of Image Processing Computer Vision and the Toolbox of Machine Learning of the MATLAB 2018a software (Mathworks), installed in an HP Z800 computer with two 2.66 GHz Xeon X5650 Intel processors with a 12 M cache, 128 Gb Ram, 6.40 GT/s Intel QPI, and a Nvidea FX 3800 video board based on the Windows 10 operational system. 

For the analysis of the deep learning algorithm, the confusion matrix was used as a metric for defining applicability which could be compared with traditional image processing algorithms. The main diagonal of the confusion matrix provides a summary of the performance of the models, since the correct classifications are found there.

Metrics derived from the confusion matrix, such as sensitivity, specificity, overall accuracy, precision and F1-score, were used to analyze the performance of the deep learning models (Table 2). In the scientific literature of machine learning, these are the main metrics adopted [49,50].

Terminology used in the table: True Positive (TP) for images correctly predicted as belonging to the target class; True Negative (TN) for images correctly predicted as not belonging to the target class; False Positive (FP) for images incorrectly predicted as belonging to the target class; and False Negative (FN) for images belonging to the target class which were predicted to belong to a different class.

Precision is the ratio of the percentage of images correctly classified for the class being analyzed to all of the elements classified as belonging to this class. Accuracy is the proportion of images correctly identified. Sensitivity or recall measures the fraction of images correctly identified as belonging to a specific class, i.e., a value which permits the identification of the class for which the method is least sensitive. Specificity is the ratio of negative samples identified correctly to the total number of negative samples. The F-score makes a balanced analysis of the system possible, independent of the weighting of the data, since it relates the correctly predicted positive examples to those correctly identified as positive (recall).

## 3. Results and Discussion

Since the objective of this paper is the analysis of deep learning models to identify lesions on cotton leaves and a comparison of these models with the performance of traditional algorithms for the processing of images, Table 3 presents a summary of the performance of the traditional image processing algorithms.

For all four algorithms, the overall accuracy was greater than 70%. These results suggest that the proposed system could utilize any one of the algorithms for the task of identifying lesions on cotton leaves [51]. However, the performance is far from ideal, since Mohanty et al. [52] obtained a figure of 99.35% for accuracy in the detection of leaf diseases; Brahimi et al. [53] found a figure of 99.18% for tomato leaves, and Zhang et al. [54], an overall accuracy greater than 90% in the identification of damage to soybeans tolerant to the herbicide dicamba. All of these values are substantially superior to those obtained here. 

On the other hand, Xu et al. [55] and Alam et al. [56] report that in systems of artificial vision for pulverization at a variable rate, even low precision values can make a contribution to a reduction in the volume of pesticides to be applied. In this situation, considering only the overall accuracy, the algorithms based on support vector machines and closest neighbors would be the most highly indicated for the development of a system for professional use. 

The RNA classifier adopted is a feedforward network with 100 neurons, trained with a feedbackward algorithm. The hybrid NFC classifier combines the characteristics of a neural network with those of fuzzy logic in an efficient way which improves the precision of recognition [37]. However, the results obtained indicate that the flexibility of the neural network and the capacity to deal with the uncertainties inherent in fuzzy logic have been improved substantially by algorithms of closest k-neighbors (9.8% better than the NFC) and of support vector machines (11.5% better).

Although not common, the use of a hybrid classifier combining a neural network with fuzzy logic for the recognition of leaf lesions via images is seen as an improvement (development), since most conventional classifiers are binary and have little or no flexibility [52]. Based on the data in the literature, which suggest considerable improvement in the performance of classifiers based on fuzzy logic and artificial neural networks in the recognition of objects and the classification of images, the NFC algorithm was expected to obtain a performance at least equivalent to that of the RNA algorithm [57,58,59].

The differences in the performance of the traditional image processing algorithms can generally be justified by the type of attribute analyzed, i.e., the descriptor of characteristics used [60]. Since in this paper, the same descriptors were used for all four algorithms, the variations in overall accuracy found here validate the arguments of Masood and Khan [61]. As well as those of Singh et al. [62], in that for the identification of symptoms of stress and disease in plants, it is the selection of the method of machine learning that is the determining factor in the success of a classification.

On the other hand, Barbedo [63] points out that the main bottlenecks for obtaining better results in rate of disease recognition in plants may be the presence of undesirable objects in the image. This is because cannot easily be separated from the region of interest (generally the leaf and stem) since symptoms are not restricted to a clearly defined area; moreover, the acquisition of images in uncontrolled conditions creates noise and differences which compromise the analysis of the image.

Another relevant issue is discussed by Krawczyk [64], who shows that unbalanced data produce biased results. Since most of the elements of a class tend to condition specialization for this class. Xiao et al. [65] explains that the main approaches for machine learning consider only the effect of the total number of samples, ignoring the degree of dispersion of the data, although this can lead to a reduction in the learning/performance.

**Figure 4:**
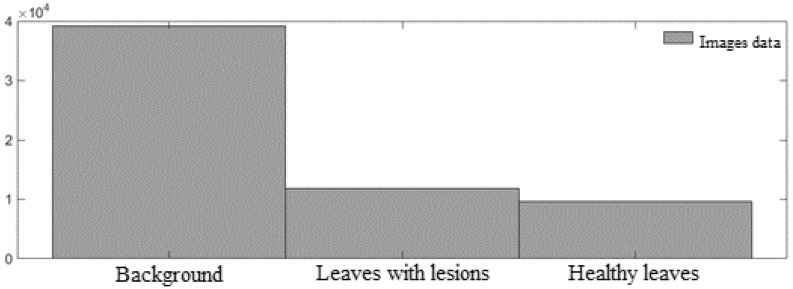
Distribution of the frequency in the original data labeled manually in the data bank of the training and testing data.

In Figure 4, the aspects pointed out by Krawczyk [64] and Xiao et al. [65] are shown to be valid, since the data utilized in the paper was unbalanced, with the background class having five times as much data as the class of lesioned leaves.

Modeling and analysis of classifiers based on unbalanced data can lead to the obtention of a tendentious model for the classification of more frequent classes [64]. According to López et al. [66]; Ramyachitra and Manikandan [67], the problem of unbalanced data may be inherent in the way classification is carried out, reflecting the high cost of obtention of data, low availability of data, or even problems in labeling arising from noise during the process of manual classification of the data.

One of the advantages of deep learning in the classification of images is that it is able to deal with an unbalanced set of data via manipulation [68]. One option is for the researcher to artificially balance the data by creating new data [69]; another is the resampling of data during the training step [70].

Here, the evaluation of the deep learning models adopted the strategy of Batuwita and Palade [48] for balancing a set of data, with resampling based on the smallest set (class) and the consequent artificial increase of all the data.

**Figure 5:**
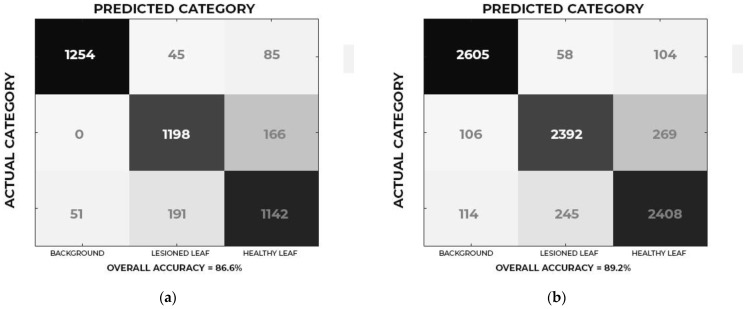
Confusion matrix of the test data for (**a**) Google Net and (**b**) ResNet50.

The confusion matrix of the deep learning models (Figure 5) shows that the technique obtained results up to 25% higher than those obtained by traditional algorithms. This reinforces the idea that models of deep learning provide reasonable improvements in the performance of classifiers [71,72,73].

**Figure 6:**
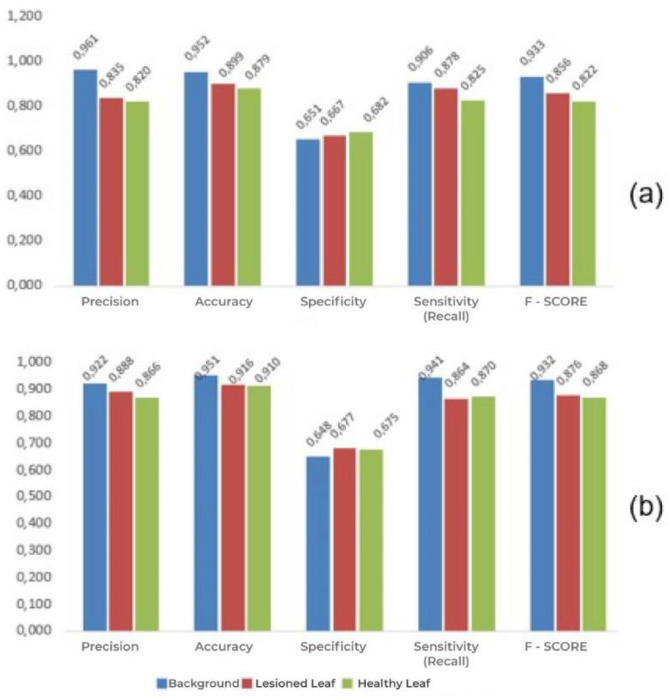
Report of the performance of the two CNN models for each class: (**a**) Google Net and (**b**) ResNet50.

In addition to the observation of the number of successes in classification in the overall context, the contribution or limitation of these for each class must be analyzed when methods of deep learning are used. Since the classes in deep learning are balanced, the best results for sensitivity (recall), specificity, accuracy, precision and F-Score for a target class define the best model. Figure 6 shows the values of the metrics of performance of the models of GoogleNet and ResNet50 for each class.

The results show that both of the models are quite satisfactory for the identification of the classes. There is a slight difference in accuracy of about 2% between lesioned leaves and healthy leaves with the use of GoogleNet CNN. For the background class, the average difference for the other classes is 14%, while with ResNet50, the difference is only 5%. In general, the average performance of the ResNet50 model is 1.67%, 2.49%, and 2.40% superior to that of GoogleNet for the variables of overall accuracy, sensitivity, and F-Score, respectively. Despite the differences, the results are quite promising when one considers visual analysis made in the field, this is characterized by the human subjectivity associated with the level of experience of the technician.

Based on the work of Zhang and Meng [74], it is believed that the results obtained can be improved if hierarchic decision-making is introduced. According to Guo et al. [75], the optimization of CNNs by means of an adaptive hierarchy can result in a precision of up to 100%. Moreover, it must be remembered that most of the approaches for the detection of leaf anomalies involve the determination of pixels, i.e., a local analysis [76,77,78] while CNNs involve the approach of image categorization, i.e., a global analysis [79].

**Figure 7:**
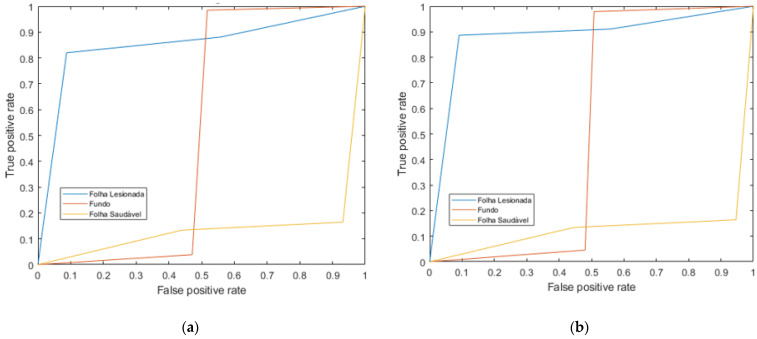
Comparison of Receiver Operating Characteristic (ROC) curves for each class for the CNN models of (**a**) GoogleNet and (**b**) ResNet50.

Finally, the Receiver Operating Characteristics (ROC) curves for the deep learning models are shown in order to evaluate the quality of the output of the classifier. Figure 7 compares the ROC curves for each of the three classes for the two models (GooleNet and ResNet50). Consistently, the images of the lesioned leaves are easiest to classify.

Since the images were captured under field conditions, i.e., with no control of lighting and exposure, as well as of being from the crops of different years and with different kinds of lesions, it is believed that the results obtained here prove the feasibility of CNN algorithms for the detection leaf lesions in the cotton crop. However, Stallkamp et al. [80] suggest that in applications of machine learning it is important to test algorithms with new data and in different situations, such as with images of other crops, wet leaves, and badly nourished plants. Such new tests should make it possible to determine the robustness and capacity of abstraction of the method developed.

## 4. Conclusions

This paper presents the results of a successful foray into the use of deep learning for the identification of lesions of cotton leaves, showing that it can indeed help in the diagnosis of agricultural pests and diseases. Two models of deep convolutional networks have thus replaced the traditional image processing in the traditional pipeline.

The main result obtained is an improvement in overall accuracy when deep learning models are used. Moreover, it has also been shown that the balancing of the data is a key to superior performance.

For both overall evaluation and the comparison between classes, the ResNet50 convolutional network proved to be more able to identify lesions; however, the average difference between its results and those of GoogleNet are insignificant from a technical point of view. Given the robustness of convolutional neural networks, we believe that the present results can be extended from the academic environment to application as operational tools.

The images for this paper were obtained in the field. They were divided into sub-images, which were processed and analyzed individually. The results reported here were obtained exclusively from this image set.

Future research will include an evaluation of the capacity of the algorithm to diagnose the causal agent of the lesion (what pest or disease). Moreover, the proposed algorithm will be implemented with the use of a software which can be utilized during actual field visits to facilitate the creation of maps of the level of infestation by pests and diseases.

## Figures and Tables

**Table 1:** Statistical texture attributes.

Characteristic	Description	Equation
I_1_	Average	$m=\overset{L-1}{\underset{i=0}{\sum }}z_{i}p\left(z_{i}\right)$
I_2_	Standard deviation	$\sigma =\sqrt{\mu_{2}\left(z\right)}=\sqrt{\sigma^{2}}$
I_3_	Smoothness	$R=1-\frac{1}{1+\sigma^{2}}$
I_4_	Third moment	$\mu_{3}=\overset{L-1}{\underset{i=0}{\sum }}{\left(z_{i-m}\right)}^{3}p\left(z_{i}\right)$
I_5_	Uniformity	$U=\overset{L-1}{\underset{i=0}{\sum }}p^{2}\left(z_{i}\right)$
I_6_	Entropy	$e=-\overset{L-1}{\underset{i=0}{\sum }}p\left(z_{i}\right)log_{2}p\left(z_{i}\right)$

**Table 2:** Performance measurements derived from the confusion matrix.

Performance Metric	Equation
Sensitivity (Recall)	TP/(TP + FN)
Specificity	TN/(TN + FP)
Overall Accuracy	(TP + TN)/(TP + FP + TN + FN)
Precision	TP/(TP + FP)
F-Score	(2 × Precision × Recall)/(Precision + Recall)

**Table 3:** Performance of classifiers using the original testing data set.

Algorithm	Overall Accuracy
SVM	80.30%
NFC	71.10%
RNA	76.60%
KNN	78.80%

## Data Availability

The data that support the findings of this study are openly available at https://drive.google.com/drive/folders/16_mK9a8mKDqyS4xalRzXp-Crnc4WZ_tr?usp=sharing (accessed on 22 April 2021).
